# Dichlorido(η^4^-cyclo­octa-1,5-diene)bis­(triphenyl­phosphine)osmium(II)

**DOI:** 10.1107/S1600536809037817

**Published:** 2009-09-26

**Authors:** Chen Ye, Ting Bin Wen

**Affiliations:** a Department of Chemistry, College of Chemistry and Chemical Engineering, Xiamen University, Xiamen 361005, Fujian, People’s Republic of China

## Abstract

The Os^II^ atom in the title compound, [OsCl_2_(C_8_H_12_)(C_18_H_15_P)_2_], is located on a crystallographic twofold axis and adopts a distorted octa­hedral coordination geometry. The two triphenyl­phosphine ligands are *trans* to each other, while the two chlorine ligands are *cis*-disposed. The coordination is completed by the cyclo­octa­diene (COD) ligand with bonding to the two olefin double bonds. The C=C bond has a length of 1.403 (6) Å, which is significntly longer than a free olefinic double bond (≃1.34 Å).

## Related literature

For general background to Ru^II^ and Os^II^ COD complexes, see: Bennett & Wilkinson (1959 ▶); Albers *et al.* (1989 ▶); Cucullu *et al.* (1999 ▶); Coalter & Caulton (2001 ▶); Alvarez *et al.* (2001 ▶); Winkhaus *et al.* (1966 ▶); Schrock *et al.* (1974 ▶); Dickinson & Girolami (2006 ▶). For C=C bond lengths for free olefinic double bonds, see: Orpen *et al.* (1989 ▶). For related COD-coordinated Os^II^ complexes, see: Esteruelas *et al.* (2006 ▶); Dickinson & Girolami (2006 ▶).
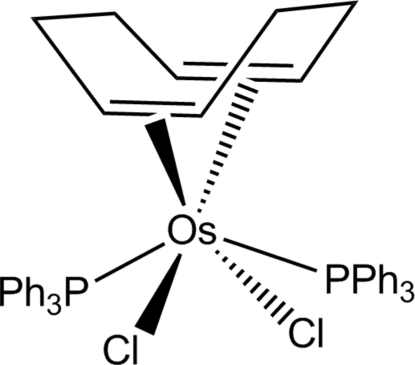


## Experimental

### 

#### Crystal data

[OsCl_2_(C_8_H_12_)(C_18_H_15_P)_2_]
*M* *_r_* = 893.82
Orthorhombic, 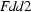

*a* = 39.6505 (15) Å
*b* = 10.4393 (5) Å
*c* = 17.6248 (8) Å
*V* = 7295.3 (6) Å^3^
*Z* = 8
Mo *K*α radiation
μ = 3.76 mm^−1^
*T* = 173 K
0.15 × 0.15 × 0.12 mm

#### Data collection

Oxford Diffraction Gemini S Ultra diffractometer
Absorption correction: multi-scan (*CrysAlis RED*; Oxford Diffraction, 2008 ▶) *T*_min_ = 0.860, *T*_max_ = 1.000
6965 measured reflections
2778 independent reflections
2546 reflections with *I* > 2σ(*I*)
*R*_int_ = 0.033

#### Refinement

*R*[*F* ^2^ > 2σ(*F* ^2^)] = 0.022
*wR*(*F* ^2^) = 0.044
*S* = 1.00
2778 reflections
222 parameters
1 restraint
H-atom parameters constrained
Δρ_max_ = 1.39 e Å^−3^
Δρ_min_ = −0.74 e Å^−3^
Absolute structure: Flack (1983 ▶), 937 Friedel pairs
Flack parameter: 0.009 (6)

### 

Data collection: *CrysAlis CCD* (Oxford Diffraction, 2008 ▶); cell refinement: *CrysAlis RED* (Oxford Diffraction, 2008 ▶); data reduction: *CrysAlis RED*; program(s) used to solve structure: *SHELXTL* (Sheldrick, 2008 ▶); program(s) used to refine structure: *SHELXTL*; molecular graphics: *SHELXTL*; software used to prepare material for publication: *SHELXTL*.

## Supplementary Material

Crystal structure: contains datablocks I, global. DOI: 10.1107/S1600536809037817/wm2258sup1.cif

Structure factors: contains datablocks I. DOI: 10.1107/S1600536809037817/wm2258Isup2.hkl

Additional supplementary materials:  crystallographic information; 3D view; checkCIF report

## Figures and Tables

**Table 1:** Selected bond lengths (Å)

Os1—C2	2.169 (5)
Os1—C1	2.195 (5)
Os1—Cl1	2.4429 (12)
Os1—P1	2.5031 (12)

**Fig. 1.**
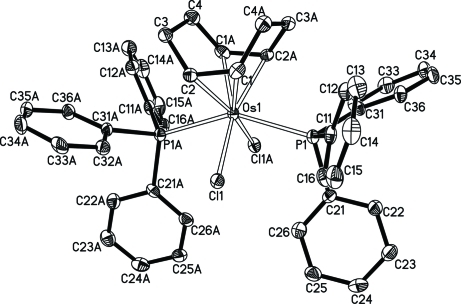
The structure of [OsCl2(C8H12)(C18H15P) 2] showing 40% probability displacement ellipsoids. H atoms have been omitted for clarity. Atoms with symmetry code letter A are related by -x + 1/2, -y + 1/2, z.

## supplementary crystallographic information

### Comment

The ruthenium polymer [RuCl_2_(COD)]*_x_* (COD = cycloocta-1,5-diene), which can be readily prepared in 30–40% yield from RuCl_3_.3H_2_O and COD in boiling ethanol (Bennett & Wilkinson, 1959; Albers *et al.*, 1989), has proved to be a useful precursor for a wide variety of ruthenium compounds (Cucullu *et al.*, 1999; Coalter & Caulton, 2001; Alvarez *et al.*, 2001). Although the analogous osmium polymer [OsCl_2_(COD)]*_x_* is also known, its utility as a starting material has remained relatively unexplored partially due to the difficuly in its preparation (Winkhaus *et al.*, 1966; Schrock *et al.*, 1974; Dickinson & Girolami, 2006). In our search for other potential precusors for the synthesis of osmium compounds, we have prepared [OsCl_2_(η^4^– COD)(PPh_3_)_2_] readily from the reaction of OsCl_2_(PPh_3_)_3_ with COD.

The title compound crystallizes in the non-centrosymmetric orthorhombic space group *Fdd*2. As shown in Fig.1, the structure possesses a crystallographic 2-fold axis passing through the osmium atom, thus the asymmetric unit contains half of a molecule. The Os^II^ centre adopts a distorted octahedral geometry with the two triphenylphosphine ligands *trans* to each other (P(1)—Os(1)—P(1 A) 148.31 (6)°), while the two chlorine ligands are *cis*-disposed (Cl(1)—Os(1)—Cl(1 A) 103.69 (6)°) (symmetry code: -*x* + 1/2, -*y* + 1/2, *z*). The coordination is completed by the two olefin double bonds of the cyclooctadiene ligand. The Os(1)—C(1) and Os(1)—C(2) bond lengths (2.195 (5) and 2.168 (5) Å, respectively) are similar to the related Os—C bond lengths reported for other COD coordinated Os^II^ complexes such as [H(EtOH)_2_[{OsCl(η^4^-COD)}_2_(µ-H)(µ-Cl)_2_] (2.129–2.152 Å) (Esteruelas *et al.*, 2006) and TpOs(η^4^-COD)OMe (Tp = trispyrazolylborate) (2.141–2.198 Å) (Dickinson & Girolami, 2006). The C(1)—C(2) (1.403 (6) Å) bond length is significntly longer than a free olefinic double bond (≈ 1.34 Å) (Orpen *et al.*, 1989) and is typical for a coordinated C═C double bond, which is also close to the C═C bond lengths found in [H(EtOH)_2_[{OsCl(η^4^-COD)}_2_(µ-H)(µ-Cl)_2_] (1.393–1.422 Å) and TpOs(η^4^-COD)OMe (1.396 (5) and 1.399 (5) Å).

### Experimental

To a solution of OsCl_2_(PPh_3_)_3_ (0.52 g, 0.50 mmol) in toluene (10 ml) under nitrogen atmosphere was added cycloocta-1,5-diene (0.20 ml, 2.5 mmol). The reaction mixture was stirred at room temperature for 30 h to give the title compound as large amount of a yellow precipitate. The solid was collected by filtration, washed with toluene (2 × 5 ml) and diethyl ether (3 × 5 ml), and dried under vaccum. Yield: 0.38 g, 85%. Crystals suitable for X-ray analysis were obtained by layering a solution of the title compound with a solution of chloroform and hexane.

### Refinement

All non-hydrogen atoms were refined anisotropically. The hydrogen atoms were positioned geometrically (C—H =0.95, 1.00 or 0.99 Å for phenyl, tertiary or methylene H atoms, respectively) and were included in the refinement in the riding model approximation. The displacement parameters of H atoms were set to 1.2*U*_eq_(C). In the final Fourier map the highest peak is 0.99 Å from atom Os1 and the deepest hole is 1.86 Å from atom C15.

### Crystal data

**Table:**

[OsCl_2_(C_8_H_12_)(PC_18_H_15_)_2_]	*F*(000) = 3568
*M**_r_* = 893.82	*D*_x_ = 1.628 Mg m^−^^3^
Orthorhombic, *F**d**d*2	Mo *K*α radiation, λ = 0.71073 Å
Hall symbol: F 2 -2d	Cell parameters from 5605 reflections
*a* = 39.6505 (15) Å	θ = 2.3–32.5°
*b* = 10.4393 (5) Å	µ = 3.76 mm^−^^1^
*c* = 17.6248 (8) Å	*T* = 173 K
*V* = 7295.3 (6) Å^3^	Block, light yellow
*Z* = 8	0.15 × 0.15 × 0.12 mm

### Data collection

**Table:**

Oxford Diffraction Gemini S Ultra diffractometer	2778 independent reflections
Radiation source: Enhance (Mo) X-ray Source	2546 reflections with *I* > 2σ(*I*)
graphite	*R*_int_ = 0.033
Detector resolution: 16.1930 pixels mm^-1^	θ_max_ = 26.0°, θ_min_ = 2.3°
ω scans	*h* = −48→48
Absorption correction: multi-scan (*CrysAlis RED*; Oxford Diffraction, 2008)	*k* = −12→12
*T*_min_ = 0.860, *T*_max_ = 1.000	*l* = −21→16
6965 measured reflections	

### Refinement

**Table:**

Refinement on *F*^2^	Secondary atom site location: difference Fourier map
Least-squares matrix: full	Hydrogen site location: inferred from neighbouring sites
*R*[*F*^2^ > 2σ(*F*^2^)] = 0.022	H-atom parameters constrained
*wR*(*F*^2^) = 0.044	*w* = 1/[σ^2^(*F*_o_^2^) + (0.0184*P*)^2^] where *P* = (*F*_o_^2^ + 2*F*_c_^2^)/3
*S* = 1.00	(Δ/σ)_max_ = 0.001
2778 reflections	Δρ_max_ = 1.39 e Å^−^^3^
222 parameters	Δρ_min_ = −0.74 e Å^−^^3^
1 restraint	Absolute structure: Flack (1983), 937 Friedel pairs
Primary atom site location: structure-invariant direct methods	Flack parameter: 0.009 (6)

### Special details

**Table:**

Geometry. All e.s.d.'s (except the e.s.d. in the dihedral angle between two l.s. planes) are estimated using the full covariance matrix. The cell e.s.d.'s are taken into account individually in the estimation of e.s.d.'s in distances, angles and torsion angles; correlations between e.s.d.'s in cell parameters are only used when they are defined by crystal symmetry. An approximate (isotropic) treatment of cell e.s.d.'s is used for estimating e.s.d.'s involving l.s. planes.
Refinement. Refinement of *F*^2^ against ALL reflections. The weighted *R*-factor *wR* and goodness of fit *S* are based on *F*^2^, conventional *R*-factors *R* are based on *F*, with *F* set to zero for negative *F*^2^. The threshold expression of *F*^2^ > σ(*F*^2^) is used only for calculating *R*-factors(gt) *etc*. and is not relevant to the choice of reflections for refinement. *R*-factors based on *F*^2^ are statistically about twice as large as those based on *F*, and *R*- factors based on ALL data will be even larger.

### Fractional atomic coordinates and isotropic or equivalent isotropic displacement parameters (Å^2^)

**Table:**

	*x*	*y*	*z*	*U*_iso_*/*U*_eq_	
Os1	0.2500	0.2500	0.134570 (16)	0.01584 (6)	
Cl1	0.27183 (3)	0.08574 (11)	0.22020 (7)	0.0225 (3)	
P1	0.19622 (3)	0.14284 (12)	0.17335 (8)	0.0183 (3)	
C1	0.25093 (11)	0.1050 (4)	0.0444 (3)	0.0232 (10)	
H1A	0.2499	0.0145	0.0629	0.028*	
C2	0.28281 (11)	0.1625 (5)	0.0509 (3)	0.0203 (10)	
H2A	0.3005	0.1053	0.0728	0.024*	
C3	0.29572 (11)	0.2541 (5)	−0.0092 (3)	0.0243 (10)	
H3A	0.2991	0.2067	−0.0572	0.029*	
H3B	0.3179	0.2882	0.0069	0.029*	
C4	0.27153 (12)	0.3668 (5)	−0.0234 (3)	0.0279 (12)	
H4A	0.2849	0.4442	−0.0357	0.033*	
H4B	0.2572	0.3468	−0.0678	0.033*	
C11	0.19375 (10)	−0.0268 (4)	0.1464 (3)	0.0222 (11)	
C12	0.18371 (11)	−0.0649 (5)	0.0742 (3)	0.0263 (12)	
H12A	0.1759	−0.0021	0.0393	0.032*	
C13	0.18481 (12)	−0.1911 (5)	0.0520 (4)	0.0342 (13)	
H13A	0.1779	−0.2156	0.0024	0.041*	
C14	0.19615 (15)	−0.2814 (6)	0.1031 (5)	0.0346 (18)	
H14A	0.1969	−0.3689	0.0885	0.041*	
C15	0.20651 (14)	−0.2471 (6)	0.1754 (5)	0.0349 (18)	
H15A	0.2142	−0.3107	0.2099	0.042*	
C16	0.20554 (11)	−0.1196 (4)	0.1973 (3)	0.0257 (12)	
H16A	0.2129	−0.0952	0.2465	0.031*	
C21	0.18731 (10)	0.1237 (4)	0.2762 (3)	0.0204 (10)	
C22	0.15626 (12)	0.0737 (5)	0.2961 (3)	0.0270 (12)	
H22A	0.1395	0.0618	0.2582	0.032*	
C23	0.14915 (12)	0.0407 (5)	0.3707 (4)	0.0318 (14)	
H23A	0.1276	0.0077	0.3835	0.038*	
C24	0.17309 (13)	0.0556 (5)	0.4255 (3)	0.0317 (13)	
H24A	0.1685	0.0310	0.4764	0.038*	
C25	0.20399 (12)	0.1068 (5)	0.4063 (3)	0.0306 (13)	
H25A	0.2207	0.1182	0.4444	0.037*	
C26	0.21088 (12)	0.1414 (5)	0.3332 (3)	0.0259 (12)	
H26A	0.2322	0.1780	0.3213	0.031*	
C31	0.15530 (10)	0.2115 (4)	0.1440 (3)	0.0191 (12)	
C32	0.14963 (12)	0.3414 (5)	0.1543 (3)	0.0240 (12)	
H32A	0.1670	0.3930	0.1754	0.029*	
C33	0.11927 (12)	0.3978 (5)	0.1344 (4)	0.0305 (13)	
H33A	0.1161	0.4873	0.1414	0.037*	
C34	0.09364 (12)	0.3237 (6)	0.1046 (3)	0.0323 (14)	
H34A	0.0731	0.3622	0.0889	0.039*	
C35	0.09823 (12)	0.1943 (6)	0.0978 (3)	0.0304 (13)	
H35A	0.0802	0.1423	0.0800	0.037*	
C36	0.12872 (12)	0.1382 (5)	0.1165 (3)	0.0246 (14)	
H36A	0.1315	0.0484	0.1106	0.029*	

### Atomic displacement parameters (Å^2^)

**Table:**

	*U*^11^	*U*^22^	*U*^33^	*U*^12^	*U*^13^	*U*^23^
Os1	0.01417 (9)	0.01427 (10)	0.01910 (11)	0.00191 (17)	0.000	0.000
Cl1	0.0193 (5)	0.0201 (6)	0.0280 (7)	0.0041 (4)	0.0005 (5)	0.0053 (6)
P1	0.0167 (5)	0.0157 (6)	0.0224 (7)	0.0016 (5)	0.0000 (5)	−0.0006 (6)
C1	0.027 (2)	0.016 (2)	0.027 (3)	0.004 (2)	0.007 (3)	−0.005 (2)
C2	0.022 (2)	0.019 (3)	0.019 (3)	−0.0009 (19)	0.000 (2)	−0.005 (2)
C3	0.023 (2)	0.027 (3)	0.023 (2)	0.001 (2)	0.000 (2)	−0.003 (3)
C4	0.030 (2)	0.026 (3)	0.028 (3)	−0.002 (2)	−0.003 (2)	0.003 (3)
C11	0.0164 (19)	0.016 (2)	0.034 (3)	−0.0028 (16)	0.004 (2)	−0.007 (2)
C12	0.024 (2)	0.023 (3)	0.031 (3)	−0.001 (2)	0.001 (2)	−0.003 (2)
C13	0.030 (3)	0.031 (3)	0.041 (3)	0.004 (2)	0.002 (3)	−0.014 (3)
C14	0.027 (3)	0.017 (3)	0.060 (5)	0.004 (2)	0.005 (3)	−0.011 (3)
C15	0.022 (3)	0.027 (3)	0.055 (5)	0.008 (2)	0.000 (3)	0.007 (4)
C16	0.021 (2)	0.020 (3)	0.035 (3)	−0.0002 (19)	−0.003 (2)	−0.001 (2)
C21	0.021 (2)	0.019 (2)	0.022 (3)	0.0058 (18)	0.005 (2)	0.005 (2)
C22	0.023 (2)	0.028 (3)	0.030 (3)	0.000 (2)	−0.001 (2)	0.005 (3)
C23	0.029 (2)	0.031 (3)	0.035 (4)	−0.0005 (18)	0.005 (3)	0.007 (3)
C24	0.043 (3)	0.029 (3)	0.024 (3)	0.008 (2)	0.004 (3)	0.005 (3)
C25	0.030 (3)	0.039 (3)	0.023 (3)	0.004 (2)	−0.002 (2)	−0.003 (3)
C26	0.025 (2)	0.025 (3)	0.028 (3)	0.004 (2)	0.004 (2)	−0.005 (2)
C31	0.0143 (18)	0.021 (3)	0.022 (4)	0.0026 (15)	0.000 (2)	−0.003 (3)
C32	0.022 (2)	0.022 (3)	0.028 (3)	0.003 (2)	0.001 (2)	−0.006 (2)
C33	0.028 (2)	0.029 (3)	0.035 (3)	0.015 (2)	0.007 (3)	−0.004 (3)
C34	0.016 (2)	0.042 (4)	0.039 (4)	0.004 (2)	0.000 (2)	0.002 (3)
C35	0.017 (2)	0.044 (3)	0.030 (3)	−0.006 (2)	−0.001 (2)	−0.010 (3)
C36	0.020 (3)	0.022 (3)	0.032 (4)	−0.003 (2)	0.001 (3)	−0.005 (3)

### Geometric parameters (Å, °)

**Table:**

Os1—C2^i^	2.169 (5)	C14—C15	1.386 (8)
Os1—C2	2.169 (5)	C14—H14A	0.9500
Os1—C1	2.195 (5)	C15—C16	1.387 (7)
Os1—C1^i^	2.195 (5)	C15—H15A	0.9500
Os1—Cl1^i^	2.4429 (12)	C16—H16A	0.9500
Os1—Cl1	2.4429 (12)	C21—C22	1.382 (6)
Os1—P1	2.5031 (12)	C21—C26	1.384 (7)
Os1—P1^i^	2.5031 (12)	C22—C23	1.387 (8)
P1—C11	1.836 (4)	C22—H22A	0.9500
P1—C31	1.848 (4)	C23—C24	1.364 (8)
P1—C21	1.858 (5)	C23—H23A	0.9500
C1—C2	1.404 (6)	C24—C25	1.379 (7)
C1—C4^i^	1.519 (7)	C24—H24A	0.9500
C1—H1A	1.0000	C25—C26	1.365 (7)
C2—C3	1.516 (7)	C25—H25A	0.9500
C2—H2A	1.0000	C26—H26A	0.9500
C3—C4	1.538 (7)	C31—C32	1.386 (7)
C3—H3A	0.9900	C31—C36	1.390 (7)
C3—H3B	0.9900	C32—C33	1.385 (7)
C4—C1^i^	1.519 (7)	C32—H32A	0.9500
C4—H4A	0.9900	C33—C34	1.381 (7)
C4—H4B	0.9900	C33—H33A	0.9500
C11—C12	1.392 (7)	C34—C35	1.368 (7)
C11—C16	1.400 (7)	C34—H34A	0.9500
C12—C13	1.375 (7)	C35—C36	1.383 (7)
C12—H12A	0.9500	C35—H35A	0.9500
C13—C14	1.380 (9)	C36—H36A	0.9500
C13—H13A	0.9500		
			
C2^i^—Os1—C2	94.3 (3)	C3—C4—H4B	109.1
C2^i^—Os1—C1	78.95 (18)	H4A—C4—H4B	107.8
C2—Os1—C1	37.52 (16)	C12—C11—C16	118.9 (4)
C2^i^—Os1—C1^i^	37.52 (16)	C12—C11—P1	121.8 (4)
C2—Os1—C1^i^	78.95 (18)	C16—C11—P1	118.9 (4)
C1—Os1—C1^i^	87.3 (3)	C13—C12—C11	121.7 (5)
C2^i^—Os1—Cl1^i^	84.95 (14)	C13—C12—H12A	119.2
C2—Os1—Cl1^i^	158.22 (12)	C11—C12—H12A	119.2
C1—Os1—Cl1^i^	159.61 (12)	C12—C13—C14	118.6 (6)
C1^i^—Os1—Cl1^i^	87.54 (13)	C12—C13—H13A	120.7
C2^i^—Os1—Cl1	158.22 (12)	C14—C13—H13A	120.7
C2—Os1—Cl1	84.95 (14)	C13—C14—C15	121.4 (6)
C1—Os1—Cl1	87.54 (13)	C13—C14—H14A	119.3
C1^i^—Os1—Cl1	159.61 (12)	C15—C14—H14A	119.3
Cl1^i^—Os1—Cl1	103.69 (6)	C14—C15—C16	119.7 (6)
C2^i^—Os1—P1	82.14 (12)	C14—C15—H15A	120.2
C2—Os1—P1	120.55 (12)	C16—C15—H15A	120.2
C1—Os1—P1	84.48 (13)	C15—C16—C11	119.7 (6)
C1^i^—Os1—P1	119.44 (12)	C15—C16—H16A	120.1
Cl1^i^—Os1—P1	80.97 (4)	C11—C16—H16A	120.1
Cl1—Os1—P1	79.60 (4)	C22—C21—C26	117.9 (5)
C2^i^—Os1—P1^i^	120.55 (12)	C22—C21—P1	117.2 (4)
C2—Os1—P1^i^	82.14 (12)	C26—C21—P1	124.4 (3)
C1—Os1—P1^i^	119.44 (12)	C21—C22—C23	121.0 (5)
C1^i^—Os1—P1^i^	84.48 (13)	C21—C22—H22A	119.5
Cl1^i^—Os1—P1^i^	79.60 (4)	C23—C22—H22A	119.5
Cl1—Os1—P1^i^	80.97 (4)	C24—C23—C22	120.1 (5)
P1—Os1—P1^i^	148.31 (6)	C24—C23—H23A	120.0
C11—P1—C31	104.8 (2)	C22—C23—H23A	120.0
C11—P1—C21	98.0 (2)	C23—C24—C25	119.3 (5)
C31—P1—C21	98.5 (2)	C23—C24—H24A	120.4
C11—P1—Os1	113.95 (14)	C25—C24—H24A	120.4
C31—P1—Os1	119.87 (15)	C26—C25—C24	120.8 (5)
C21—P1—Os1	118.45 (15)	C26—C25—H25A	119.6
C2—C1—C4^i^	120.5 (4)	C24—C25—H25A	119.6
C2—C1—Os1	70.2 (3)	C25—C26—C21	120.9 (4)
C4^i^—C1—Os1	115.2 (3)	C25—C26—H26A	119.5
C2—C1—H1A	114.5	C21—C26—H26A	119.5
C4^i^—C1—H1A	114.5	C32—C31—C36	117.5 (4)
Os1—C1—H1A	114.5	C32—C31—P1	119.0 (4)
C1—C2—C3	121.2 (4)	C36—C31—P1	123.3 (4)
C1—C2—Os1	72.3 (3)	C31—C32—C33	121.6 (5)
C3—C2—Os1	114.3 (3)	C31—C32—H32A	119.2
C1—C2—H2A	114.2	C33—C32—H32A	119.2
C3—C2—H2A	114.2	C34—C33—C32	119.8 (5)
Os1—C2—H2A	114.2	C34—C33—H33A	120.1
C2—C3—C4	112.7 (4)	C32—C33—H33A	120.1
C2—C3—H3A	109.1	C35—C34—C33	119.2 (5)
C4—C3—H3A	109.1	C35—C34—H34A	120.4
C2—C3—H3B	109.1	C33—C34—H34A	120.4
C4—C3—H3B	109.1	C34—C35—C36	120.9 (5)
H3A—C3—H3B	107.8	C34—C35—H35A	119.5
C1^i^—C4—C3	112.7 (4)	C36—C35—H35A	119.5
C1^i^—C4—H4A	109.1	C35—C36—C31	120.8 (5)
C3—C4—H4A	109.1	C35—C36—H36A	119.6
C1^i^—C4—H4B	109.1	C31—C36—H36A	119.6

Symmetry codes: (i) −*x*+1/2, −*y*+1/2, *z*.
